# FACT Assists Base Excision Repair by Boosting the Remodeling Activity of RSC

**DOI:** 10.1371/journal.pgen.1006221

**Published:** 2016-07-28

**Authors:** John Lalith Charles Richard, Manu Shubhdarshan Shukla, Hervé Menoni, Khalid Ouararhni, Imtiaz Nisar Lone, Yohan Roulland, Christophe Papin, Elsa Ben Simon, Tapas Kundu, Ali Hamiche, Dimitar Angelov, Stefan Dimitrov

**Affiliations:** 1 Université Joseph Fourier-Grenoble 1, INSERM Institut Albert Bonniot U823, Site Santé, Grenoble, France; 2 Université de Lyon, Laboratoire de Biologie Moléculaire de la Cellule, LBMC CNRS/ENSL/UCBL UMR5239 & Institut NeuroMyoGène–INMG CNRS/UCBL UMR5310, Ecole Normale Supérieure de Lyon, Lyon, France; 3 Institut de Génétique et de Biologie Moléculaire et Cellulaire, CNRS/INSERM/ULP, Parc d’Innovation, Illkirch, France; 4 Transcription and Disease Laboratory, Molecular Biology and Genetics Unit Jawaharlal Nehru Centre for Advanced Scientific Research, Bangalore, India; DIBIT, Milano, ITALY

## Abstract

FACT, in addition to its role in transcription, is likely implicated in both transcription-coupled nucleotide excision repair and DNA double strand break repair. Here, we present evidence that FACT could be directly involved in Base Excision Repair and elucidate the chromatin remodeling mechanisms of FACT during BER. We found that, upon oxidative stress, FACT is released from transcription related protein complexes to get associated with repair proteins and chromatin remodelers from the SWI/SNF family. We also showed the rapid recruitment of FACT to the site of damage, coincident with the glycosylase OGG1, upon the local generation of oxidized DNA. Interestingly, FACT facilitates uracil-DNA glycosylase in the removal of uracil from nucleosomal DNA thanks to an enhancement in the remodeling activity of RSC. This discloses a novel property of FACT wherein it has a co-remodeling activity and strongly enhances the remodeling capacity of the chromatin remodelers. Altogether, our data suggest that FACT may acts in concert with RSC to facilitate excision of DNA lesions during the initial step of BER.

## Author Summary

In the nucleus, DNA is packaged into chromatin. The repeating unit of chromatin, the nucleosome, consists of a histone octamer around which DNA is wrapped into two superhelical turns. The nucleosome is a barrier for various nuclear processes which require access to DNA. To repair lesions on DNA, this barrier has to be overcome by either nucleosome remodeling or by histone eviction. Here we present evidence that FACT, a protein known to be involved in transcription, is also involved in BER, by boosting nucleosome remodeling. Upon *in vivo* oxidized DNA lesion induction, FACT exhibits a BER-like protein behavior, and it is recruited to the sites of DNA damages. *In vitro* experiments show that FACT boosts the remodeling activity of the chromatin remodeler RSC and is implicated in DNA repair. The presence of FACT greatly facilitates the removal of uracil from nucleosomal, but not from naked DNA, in a RSC-mediated reaction. Taken collectively, our *in vitro* and *in vivo* data reveal a role of FACT in BER by helping the remodeling of chromatin at the sites of lesions.

## Introduction

DNA is packaged under the form of chromatin in the eukaryotic nucleus. The nucleosome, the repeating unit of chromatin, consists of an octamer of core histones (two each of H2A, H2B, H3 and H4), around which DNA is wrapped in two superhelical turns [1]. Linker DNA, connecting the consecutive nucleosomes, interacts with a fifth histone H1, termed linker histone, [1]. Each core histone contains a structured domain, the histone fold, and an unstructured NH_2_-terminus [2–4]. Both the linker histone and the NH_2_-termini of core histones are involved in the assembly and maintenance of the 30 nm chromatin [5–7] fiber and the mitotic chromosome [8,9].

The nucleosome is a barrier for several processes requiring access to the naked DNA underlying sequences [10,11]. The three main strategies that the cell uses to overcome the nucleosome barrier are the posttranslational modifications of core histones [12], the incorporation of histone variants in chromatin [13,14] and the chromatin remodelers [15,16]. The chromatin remodelers are very sophisticated nanomachines able to perturb the histone-DNA interactions and to mobilize the histone octamer along DNA by using the energy freed by the hydrolysis of ATP [15–17,18,19]. Depending on the type of ATPase present in the complex, they are classified in at least four main groups, namely the SWI/SNF, ISWI, CHD and INO80 families [20]. The chromatin remodeler RSC belongs to the SWI/SNF family and it is involved in the repair of damaged DNA [21,22]. RSC contains a central cavity sufficient for binding of a single nucleosome [23]. We have recently analyzed the mechanism of RSC-induced nucleosome mobilization and have shown that RSC generates initially an ensemble of particles with highly altered histone-DNA interactions, which are further mobilized by RSC [24].

Base Excision Repair (BER) is the major pathway to repair highly mutagenic lesions present in cellular DNA due to inherent instability of some nucleobases or induced upon oxidative stress (e.g. uracil and 8-oxo-7,8-dihydroguanine (8-oxoG)). The molecular mechanisms of BER on naked DNA are well understood, but how it functions on chromatin templates remains elusive. The reported data show that the presence of nucleosomes interferes strongly with BER, although the different enzymes are affected in a distinct manner [25–29]. For instance, within nucleosomal DNA, the activity of uracil DNA glycosylase (UDG) on uracil facing the solvent is only slightly reduced, while the removal of uracil facing the histone octamer is greatly (up to 3–4 orders of magnitude) inhibited [30]. In addition, the very strong inhibition of 8-oxoG removal by the glycosylase OGG1 was overcome in the presence of nucleosome remodeling complexes [31,32].

Human FACT (Facilitates Chromatin Transcription) protein consists of two subunits, hSpt16 and SSRP1, which are both required for its functionality [33]. FACT exhibits a histone chaperone activity; it makes a complex with the H2A-H2B dimer and the (H3-H4)_2_ tetramer and is able to deposit them on DNA [33,34]. Few reports indicate that, in addition to its role in transcription, FACT might be implicated in the Transcription-Coupled Nucleotide Excision Repair (TC- NER) of UVC-damaged DNA or DNA Double Strand Break (DSB) repair [35–38]. FACT was found in a complex with casein kinase 2 (CK2), known to phosphorylate p53 in a DNA damage dependent manner resulting in an increase of p53 activity [35,36]. FACT appears also to be involved in both phosphorylation and exchange of histone variant H2A.X, two events related to the DSB repair [37].

In this work we show that upon oxidatively induction of DNA damage, FACT switches protein partners from transcription-associated factors to DNA repair protein. FACT together with chromatin remodelers from the SWI/SNF family is recruited to the sites of DNA damage. This DNA damage response strongly suggests an implication of FACT in BER. To shed light on the mechanism of FACT in this process, we have carried out a series of *in vitro* experiments using uracil containing nucleosomes. We reveal that FACT greatly facilitates the removal of uracil by UDG from the nucleosomal DNA *via* a novel mechanism, implicating boosting of the activity of the ATP-dependent chromatin remodeler RSC. Collectively, our data points towards a role of FACT in *in vivo* BER of DNA lesions though assisting the remodeling of chromatin at the sites of lesions.

## Results

### FACT is recruited to the sites of oxidative DNA damage

**Fig 1:**
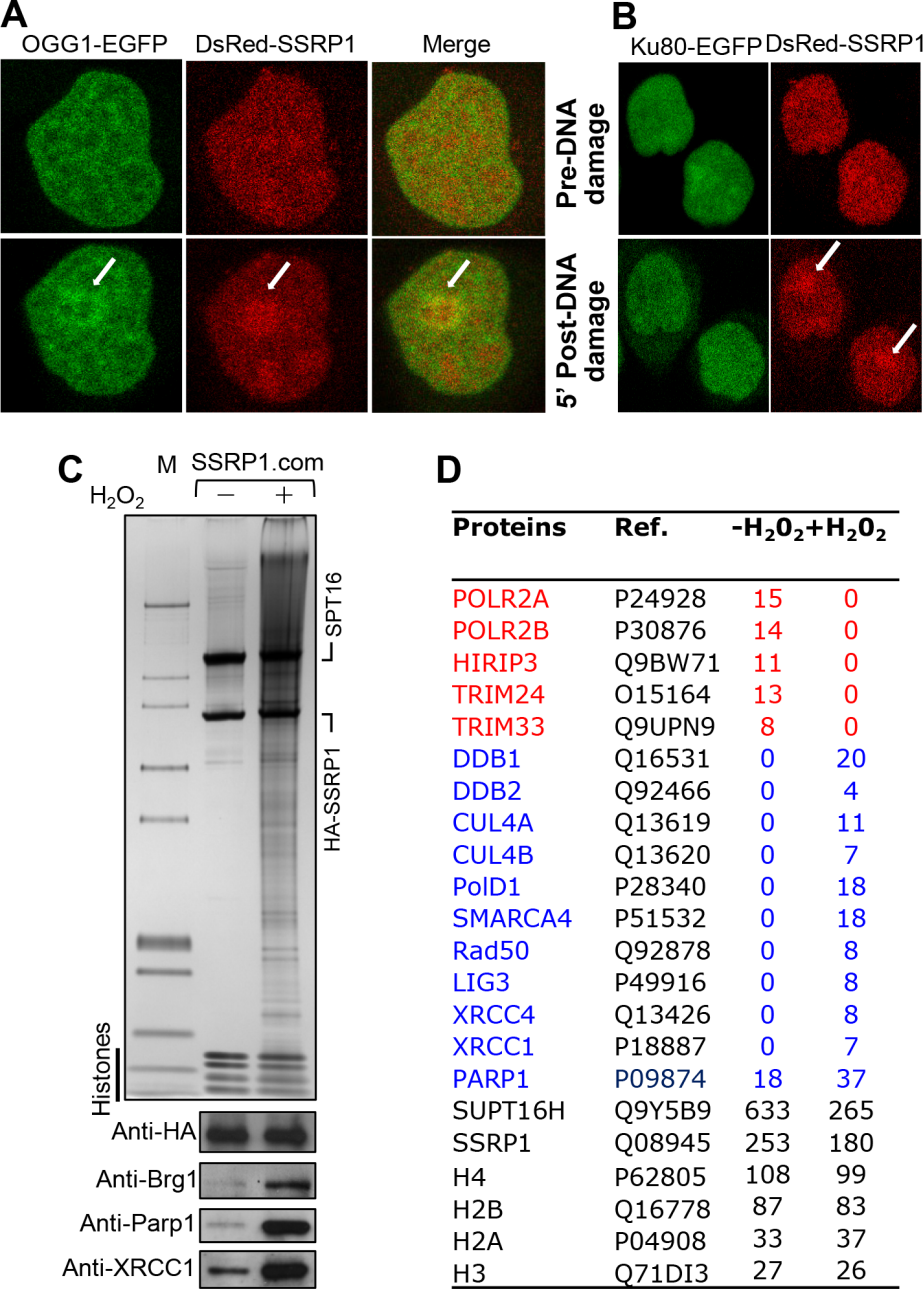
FACT is implicated in the repair of oxidatively damaged DNA. (**A**) FACT is recruited to the sites of oxidative DNA lesions. HeLa cells expressing either OGG1-EGFP (left) or DsRed-SSRP1 (the smaller subunit of FACT, right) were locally irradiated with 405-nm laser in the absence (upper two panels) or presence (lower two panels) of photosensitizer Ro-19-8022 and the accumulation of the fusions at the bleached sites (indicated with white arrow) was observed 5 minutes post irradiation (lower panels). (**B**) Same as (A), but for Hela cells expressing either Ku80-EGFP or DsRed-SSRP1. (**C**) Treatment of cells with H_2_O_2_ results in release of FACT from transcribed chromatin and in binding to chromatin regions associated with both DNA repair proteins and chromatin remodeling factors. Stable Hela cell lines expressing a fusion of HA with SSRP1, treated or not with H_2_O_2_, were used to immunopurify the chromatin bound FACT complexes. Upper panel shows the silver stained SDS gel of the proteins associated with either control FACT chromatin bound complex (-) or with the FACT chromatin bound complex isolated from H_2_O_2_ treated cells (+). The lower panel shows the association of the indicated proteins identified by Western blotting in the respective complexes. (**D**) Mass spectrometry identification of the polypeptides associated with control FACT chromatin bound complex (-) or with the FACT chromatin bound complex, isolated from H_2_O_2_ treated cells (+). Proteins present in the e-SSRP1.com together with the number of identified peptides are indicated. Proteins involved in transcription are shown in red. DNA repair proteins and chromatin remodelers are shown in blue.

Some available data indicate that FACT, in addition to transcription, is involved, as mentioned above, in both transcription-coupled nucleotide excision repair (TC-NER) and DNA double strand break (DSB) repair [35–38]. However, no data is available on the implication of FACT in BER. If FACT is directly implicated in the repair of oxidatively damaged DNA it should be recruited to the sites of induced lesions in DNA. To test if FACT is recruited at BER-lesions we used HeLa cells transiently expressing fluorescently labeled FACT, i.e. a fusion of DsRed with SSRP1 (DsRed-SSRP1), the smaller subunit of FACT. As controls, HeLa cells expressing fluorescently labeled either the glycosylase OGG1 (EGFP-OGG1) or the DSB repair factor Ku80 (Ku80-GFP) were used. These cells were treated as in [39] with Ro 19–8022, a specific type II photosensitizer generating exclusively 8-oxoG [40,41] in a region microirradiated with low intensity 405 nm laser light. Upon microirradiation, DsRed-SSRP1, similarly to the repair fusion EGFP-OGG1, was rapidly recruited to the site of 8-oxoG (Fig 1A). As expected, no recruitment of the Ku-80 protein was detected (Fig 1B), thus revealing the absence of DSB generation under our irradiation conditions. Note, that our control experiments with shRNA FACT KD cells showed that the absence of FACT does not abolish OGG1 recruitment to the micro-damaged foci, as the low signal-to-noise ratio and statistical signal fluctuations did not allowed a reliable quantitative comparison. Therefore, FACT co-localizes with BER protein suggesting it might be implicated in the repair of oxidatively generated DNA lesions.

To further consolidate this hypothesis we have carried out a series of biochemical experiments. Briefly, we generated stable HeLa cell lines expressing HA-tagged SSRP1 (HA-SSRP1) and treated them with 10 mM H_2_O_2_ for 5 min at 4°C to induce oxidative damage to DNA. Then we isolated the chromatin-associated HA-SSRP1 complex (HA-SSRP1.com) from both control (non-H_2_O_2_ treated) and H_2_O_2_-treated cells by immuno-affinity using anti-HA antibody. Both complexes were next run on SDS gels and the associated polypeptides were identified by mass spectrometry and Western blotting (Fig 1C and 1D). As seen (Fig 1D), the HA-SSRP1 complex, isolated from H_2_O_2_-treated HeLa cells, contains many additional polypeptides compared to the one isolated from the control untreated cells. Mass spectrometry analysis shows that a large number of proteins involved in repair of damaged DNA (DDB1, DDB2, XRCC1, CUL1, MRE11A, XRCC4, PARP-1, etc.) as well as SMARCA1, SMARCA4 and SMARCC2 proteins, subunits of the human SWI/SNF family remodelers, co-immunopurify with the HA-SSRP1 complex, isolated from H_2_O_2_ treated HeLa cells (see Fig 1C and 1D and S1 Table). Note that these proteins were not found associated with the HA-SSRP1 complex purified from the control HeLa cells (Fig 1C and 1D and S1 Table). While HA-SSRP1, in control cells, interact with proteins involved in transcriptional control (PolR2A, PolR2B, PAF1, HIRIP3, TRIM24, TRIM33), HA-SSRP1 complex, isolated from the DNA damaged cells, (Fig 1B and 1C and S1 Table) are devoid to these proteins. Western blotting analysis for some of the proteins provided further support to this finding (Fig 1D, bottom panel). Thus, induction of DNA damages results in release of FACT associated with Pol II containing chromatin. This is accompanied with recruitment of both FACT and repair proteins to the damage sites. Note that chromatin remodelers of the SWI/SNF family are known to be implicated in DNA repair [31,32,42]. Taken as a whole, this set of data suggests that FACT participates directly in the repair of oxidative DNA lesions.

### Effect of FACT on UDG removal of uracil from nucleosomal DNA

**Fig 2:**
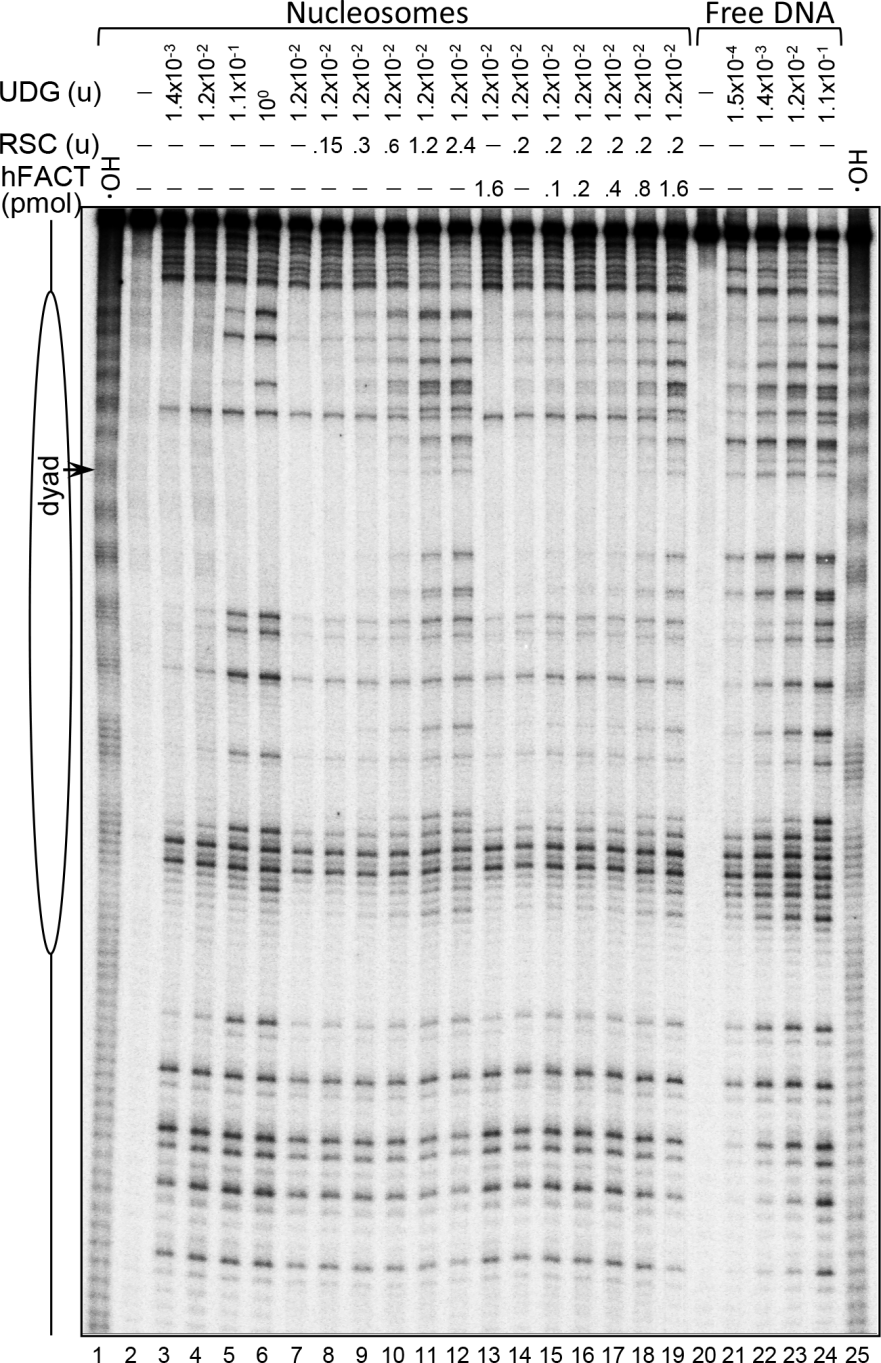
The simultaneous presence of both FACT and RSC, but not FACT alone, is required for efficient UDG excision of uracil at nucleosomal DNA sites oriented towards the histone octamer. Centrally positioned nucleosomes were reconstituted by using ^32^P 5’-labeled 255 bp 601 DNA fragment containing randomly incorporated uracil residues. **Lanes 1–6**: analysis of the UDG enzymatic activity within the nucleosomal DNA. The nucleosome solution was incubated with the indicated increasing (nine-fold step) amount of UDG for 60 minutes at 30°C and the cleavage pattern of the isolated DNA was analyzed using PAGE under denaturing conditions; lane 2: no UDG added; lanes 1, 25: •OH footprinting of the nucleosomes. **Lanes 7–12**: RSC induces a highly efficient UDG-mediated excision of uracil at inward facing sites within the nucleosome. Nucleosomes were incubated with increasing (two-fold step) amount of RSC (units) for 50 min at 30°C, and after arresting the reaction they were treated with 1.2x10^-2^ units of UDG and the isolated cleaved DNA analyzed on denaturing PAGE; lane 7, control with no RSC added in the reaction. **Lanes 13–19**: FACT facilitates the RSC-dependent UDG excision of uracil at inward facing sites within the nucleosome. Nucleosomes were incubated with increasing (2-fold step) amount of FACT in the presence of 0.2 units of RSC and, after arresting the reaction they were treated with 1.2x10^-2^ units of UDG. The cleaved purified DNA was analyzed on denaturing PAGE; lane 13, control containing 1.6 pmol of FACT with no RSC added. Note that the excision of uracil by UDG is unaffected at this highest concentration of FACT used in the experiment. **Lanes 20–24**: UDG cleavage pattern of the naked 255 bp 601 fragment. The experiment was carried out as described in Lines 2–6, but with nine-fold smaller concentration of UDG on each respective point; on the left is shown schematics of the nucleosome.

**Fig 3:**
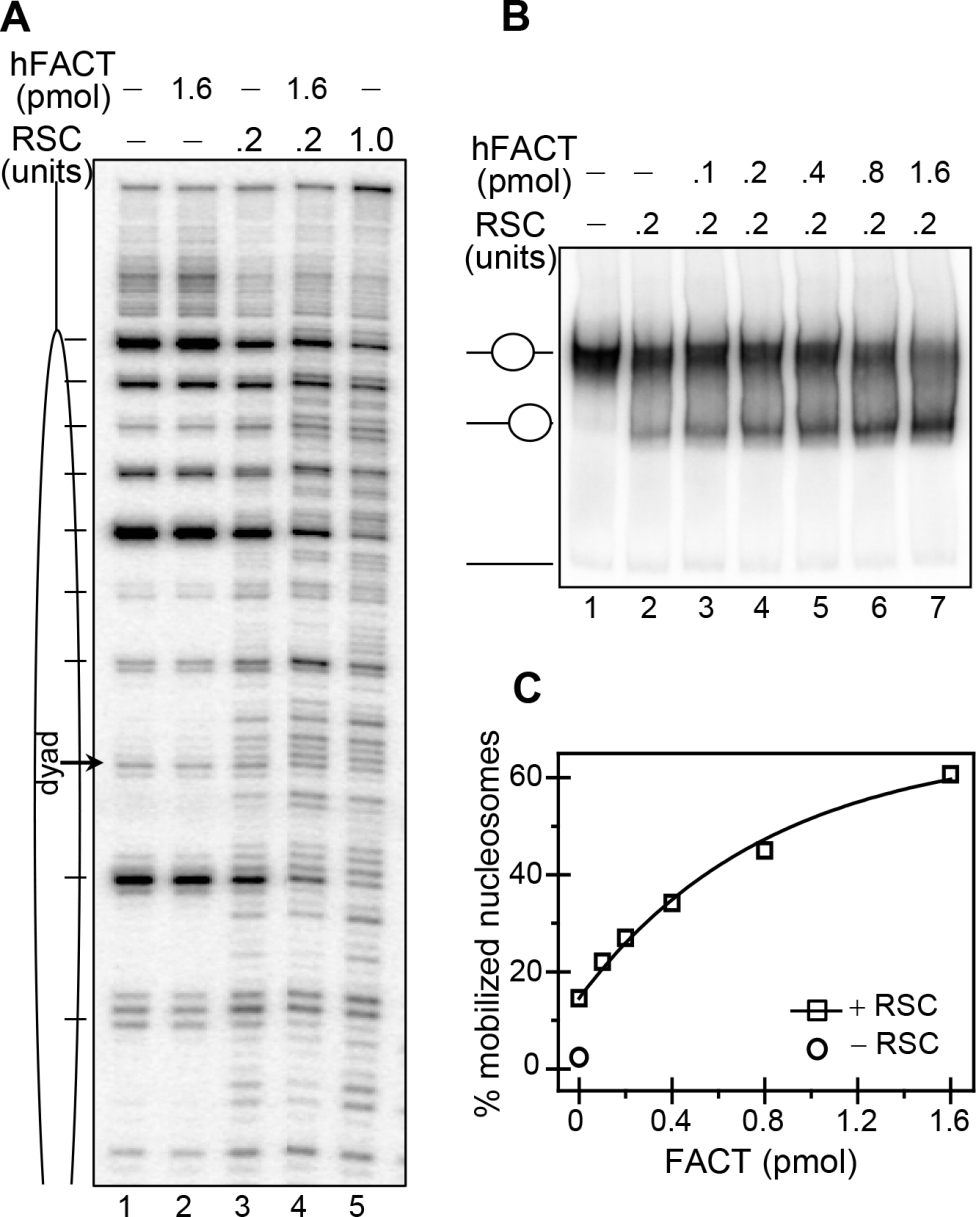
FACT facilitates both RSC-induced remodeling and mobilization of nucleosomes. (**A)** DNase I footprinting. End-positioned nucleosomes, reconstituted on ^32^P 5’-labeled 241 bp 601 DNA fragment, were incubated with 0.2 units of RSC in the absence (lane 3) or in the presence of 1.6 pmol of FACT (lane 4) for 50 min at 30°C; lane 5, same as lane 3, but with 1 unit of RSC; After arresting the remodeling reaction, the samples were digested with 0.1 units of DNase I for 2 min, the cleaved DNA was isolated and run on 8% PAGE under denaturing conditions; lanes 1 and 2, controls showing the DNase I cleavage pattern of nucleosomes (lane 1) alone or incubated with 1.6 pmol FACT under the conditions described above. (**B**) The presence of FACT increases the efficiency of RSC-induced nucleosome mobilization. Centrally positioned nucleosomes were incubated with 0.2 units of RSC in the presence of increasing amount of FACT, the reaction was arrested and the samples were run on native PAGE. The position of the non-mobilized and the slid end-positioned nucleosomes were indicated; lane 1 control nucleosomes; lane 2, nucleosomes incubated with RSC alone (in the absence of FACT). (**C**) Quantification of the data presented in (B).

How could FACT function in the repair of oxidatively damaged DNA? To address this, we focused on the role of FACT in BER and analyzed the effect of FACT on UDG removal of uracil from nucleosomal DNA using *in vitro* reconstituted centrally positioned nucleosomes. FACT was purified to homogeneity from HeLa cells by double immuno-affinity procedure (S1C Fig and Materials and Methods). The histone octamer was assembled with highly purified recombinant core histones (S1A Fig) and the nucleosome reconstitution was carried out by using the 601 nucleosome positioning sequence, containing randomly incorporated uracil [30]. The reconstitution conditions were optimized and essentially all DNA was assembled into nucleosomes (S1B Fig). The •OH and DNase I footprintings (Figs 2 and 3) showed clear 10 bp cleavage pattern further confirming the proper wrapping and the strong translational-rotational positioning of the nucleosomal DNA around the histone octamer in the reconstituted samples.

The reconstituted nucleosome samples were then incubated with increasing amount of UDG (Fig 2, lanes 3–6), DNA was isolated and, after APE1 treatment (to cleave the DNA phosphodiester bond at the abasic sites generated upon removal of uracil by UDG), the cleaved DNA was run on a PAGE under denaturing conditions. The cleavage patterns of linker DNA within nucleosomal and naked DNA are identical (Fig 2, compare lanes 3–6 with lanes 21–24). By contrast, the cleavage pattern of the histone interacting nucleosome core DNA strongly differed from the respective regions within naked DNA (compare lanes 3–6 with lanes 21–24). Cleavage in nucleosome core DNA is only observed at the sites facing the solution, which are also accessible to •OH radicals (lanes 1 and 25) even at the highest concentration of UDG used (lane 6). This is in perfect agreement with the reported data [30,43] and illustrates the inability of UDG to remove uracil from sites facing the histone octamer. Noteworthy, the presence of 1.6 pmol FACT in the reaction mixture does affect neither the efficiency nor the pattern of removal of uracil by UDG (compare lane 13 with lane 4; note that in both cases the same concentration of UDG (1.2x10^-2^ units) was used).

Pretreatment of the nucleosome samples with increasing amount of RSC changed completely the UDG cleavage pattern of nucleosomal DNA, i.e. it became qualitatively indistinguishable from that of naked DNA (compare lanes 8–12 with lanes 21–24). Thus, the RSC induced remodeling of the nucleosomes renders all uracil residues (including the ones facing the histone octamer) accessible to UDG. Interestingly, the same effect was observed when only 0.15 units of RSC (an amount of RSC unable to change the UDG accessibility to uracil, see lane 8) and increasing amount of FACT was used for the pretreatment of nucleosomes (compare lanes 14–19 with lanes 21–24). This shows that *in vitro* FACT and RSC act in concert to facilitate the UDG removal of uracil from nucleosomal DNA and suggests that *in vivo* they could act similarly to assist BER.

### FACT boosts both the remodeling activity and the capacity of RSC to mobilize the nucleosomes

Bearing in mind the above described results, we hypothesized that FACT facilitates UDG accessibility to uracil in nucleosomal DNA by acting on the remodeling activity of RSC, as it was found for nucleolin [44]. To address this we have used DNase I footprinting. Briefly, we incubated 601 end-positioned nucleosomes with RSC either in the absence or presence of FACT and after arresting the remodeling reaction, the samples were treated in controlled manner with DNase I. The cleaved DNA was then purified and analyzed on a PAGE under denaturing conditions (Fig 3A). The presence of FACT alone, as expected, did not change the clear 10 bp cleavage pattern of nucleosomal DNA (Fig 3A, compare lane 1 with lane 2). So, under the conditions of the experiment, FACT does not alter the structure of the nucleosome. When incubated with 0.2 units of RSC, the nucleosomes exhibited some alterations in the DNase I cleavage pattern, testifying for some relatively small perturbations in the histone-DNA interactions induced by RSC (compare lane 3 with lanes 1 and 2). Remarkably, the treatment of the nucleosomes with the same amount of RSC, but in the presence of 1.6 pmol of FACT, resulted in pronounced alterations in the DNase I digestion pattern (lane 4). The same altered DNase I digestion pattern was observed with 5-fold more RSC in the absence of FACT (1 unit, lane 5). We concluded that FACT exhibits strong “co-remodeling” activity and is able to boost no less than 5-fold the remodeling activity of RSC.

The effect of FACT on the nucleosome mobilization efficiency of RSC was studied by EMSA (Fig 3B and 3C). Treatment with 0.2 units of RSC led to mobilization of a very small part of the nucleosomes (not exceeding 15%, see Fig 3B and quantification on Fig 3C). The presence of increasing amount of FACT in the reaction mixture led to a strong increase in the amount of slid end–positioned nucleosomes and already at the highest concentration (1.6 pmol) of FACT, the slid nucleosomes represent ∼60% of the overall nucleosome population (Fig 3C). Similarly, the presence of FACT in the remodeling mixture led to strong increase in the time-course of the mobilization reaction (S2 Fig). Therefore, FACT boosts the RSC ability to mobilize the nucleosomes.

### FACT efficiently assists RSC to generate nucleosome-like structures exhibiting high accessibility to restriction enzyme

Our high-resolution microscopy and biochemical data show an intriguing behavior of RSC nucleosome remodeling. This consists of a formation of RSC released stable, non-mobilized particles, termed remosomes [24], which contain ∼180–190 bp of DNA loosely attached to the histone octamer [45]. The remosomes are formed by RSC-pumping ∼15–20 bp from each end of the free DNA linkers of the nucleosome without repositioning of the histone octamer. Subsequently, the remosomes are mobilized by RSC within a second interaction step [24]. Note that these “remosomes” are quite different from the Kornberg’s “altered nucleosomes” [46] and Kingston’s “novel band” [47] induced at a very high enzyme/nucleosome ratio ~0.5–2 by RSC or SWI/SNF, respectively, and identified as nucleosomal dimers. Interestingly, under our experimental conditions of enzyme/nucleosome ratio ~1/20–1/100, we did not observe any histone eviction from remodeled nucleosomes, by contrasts to reported excessive dimer (octamer) eviction after sliding (see [48] and references therein). Most likely the later observations, occurring at very high enzyme/substrate ratio ~0.5–1, represents nucleosomal DNA border (limited DNA length) artefacts of excessively “over-mobilized” mono- (di-, tri-) nucleosomes leading to unstable non-canonical (dyadless) nucleosomal species described in [49], that can lose histones due to excessive binding-dissociation events of the remodeler and/or interaction with surrounding histone acceptors.

**Fig 4:**
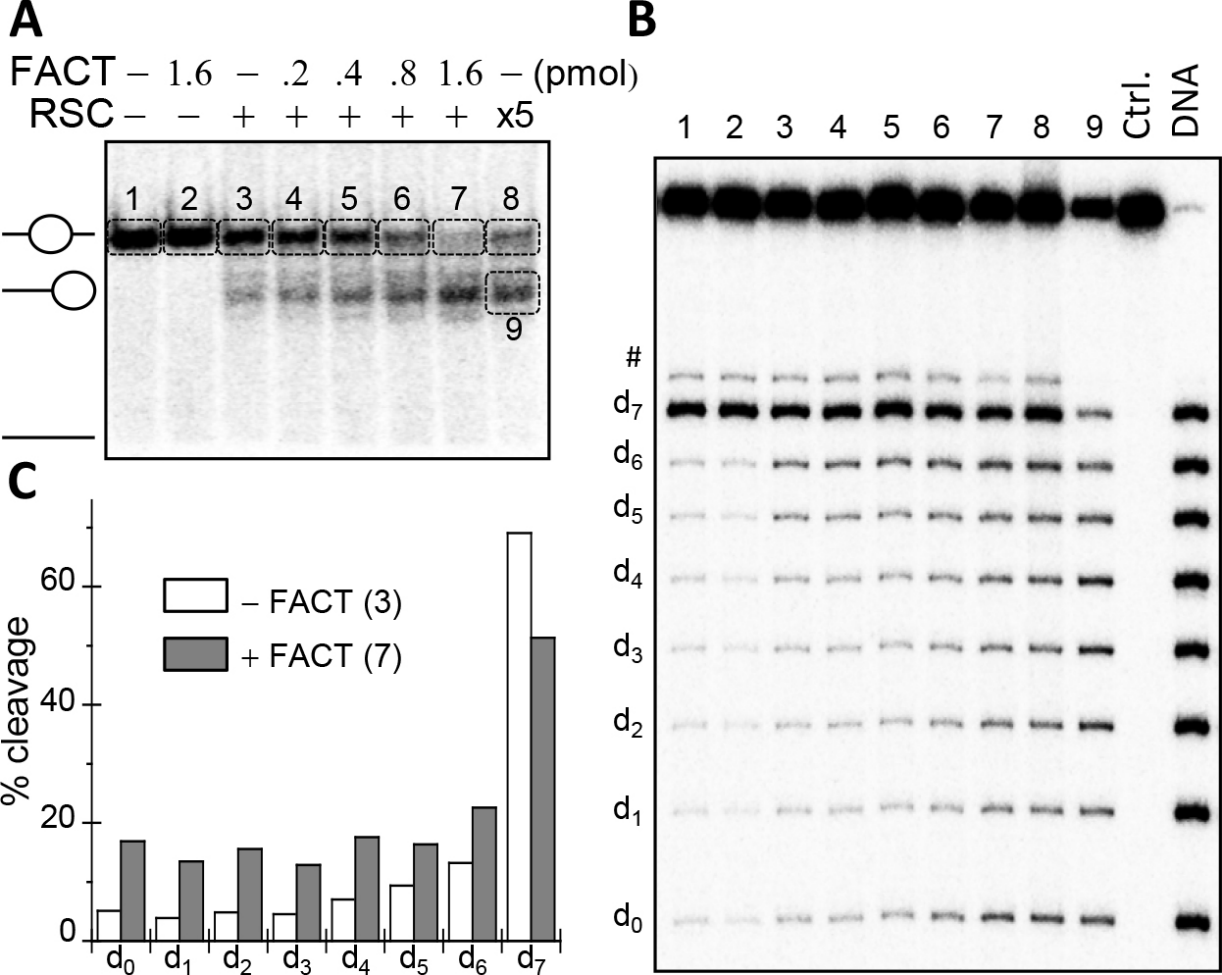
“In gel one pot assay” analysis of the effect of FACT on the DNA accessibility towards *Hae*III along the length of nucleosomal DNA in control and RSC treated nucleosomes. (**A, B**) Effect of FACT on RSC-induced remosomes generation. (**B**) Preparative PAGE. Centrally positioned nucleosomes were treated with increasing amount of FACT in the presence of 0.2 units of RSC and after arresting the reaction they were separated on native PAGE; last lane, nucleosomes treated with 5-fold higher amount (1 unit) of RSC, in the absence of FACT; the first three lanes, untreated, and treated with FACT and with 0.2 units of RSC nucleosomes, respectively. The indicated bands (from 1 to 9) were excised from the gel and in-gel digested with 8 units of *Hae*III for 10 minutes at 30°C. The cleaved DNA was then isolated and separated in 8% PAGE under denaturing conditions (**B**). The positions at the cleavage of the different dyads are indicated on the left; the numbers of each lane refers to the respective excised bands from the preparative PAGE (see A); ctrl, control, non-digested DNA; DNA, naked DNA used for reconstitutions of the nucleosomes digested with *Hae*III. (**C**) Quantification of the data presented in (B).

The main characteristic of the remosome is the higher accessibility of its DNA to restriction enzymes. To test if FACT was able to modify distinct compartments of RSC induced nucleosome remodeling, we have used the recently developed “in gel one pot assay” [24]. This approach detects quantitatively the alterations in histone-DNA interactions with a 10 bp resolution all along the nucleosomal DNA (Fig 4 and [24,50]). Briefly, eight mutated ^32^P-end labeled 255 bp 601.2 sequences were used to reconstitute centrally positioned nucleosomes. A single *Hae*III restriction site (designated as d_0_ to d_7_, where the subscript refers to the number of helical turns from the nucleosome dyad) was inserted within each of these sequences. An equimolar mixture of the eight reconstituted nucleosomes was incubated with appropriate amount of RSC either alone (to produce 10–15% of slid nucleosomes) or in the presence of increasing amount of FACT (Fig 4A). A FACT-concentration dependent mobilization of the nucleosome is observed as judged by the EMSA (Fig 4A). The upper electrophoretic band, containing the remosome fraction as well as the designated end-positioned slid nucleosome fraction were excised and in gel digested with *Hae*III. The digested DNA was purified from the gel and run on an 8% PAGE under denaturing conditions. A similar experiment using either control nucleosomes or nucleosomes treated with FACT (in the absence of RSC) was also performed. As seen (Fig 4B and 4C), and in agreement with the reported data [24], the restriction enzyme *Hae*III accessibility of the control particles at d_6_ to d_0_ is very low and practically unaffected by the presence of FACT. This agrees with the available data [50] and reveals that FACT alone, in the concentrations used, does not destabilize the histone-DNA interactions within the nucleosome.

However, upon incubation with both RSC and FACT, DNA exhibited highly altered accessibility all along the nucleosome (Fig 4B and 4C). The accessibility of d_7_ decreased relative to the control particles (this effect is due to the “pumping” of linker DNA in the nucleosome), while that of the other positions strongly increased. This increase in the *Hae*III accessibility profile is concomitant with the increase of FACT concentration used in the remodeling reaction. Since this altered *Hae*III accessibility profile is a remosome specific structural “signature” [24], we conclude that FACT assists RSC in perturbing the histone-DNA interactions in the nucleosome, while maintaining the distinct compartments of remodeling.

### UDG removes histone octamer facing uracil from both remosomes and slid nucleosomes with the same efficiency

**Fig 5:**
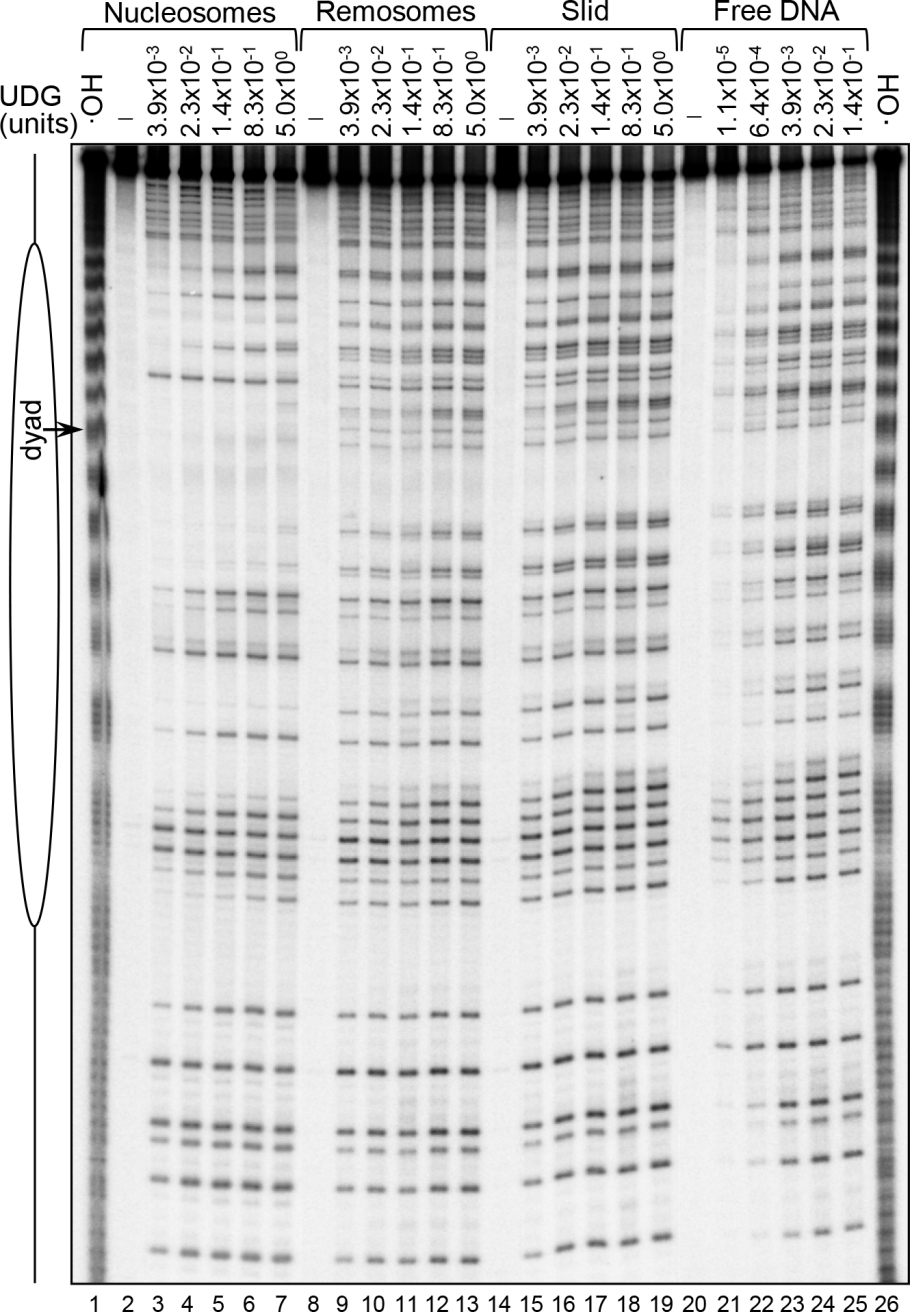
Efficient UDG excision of uracil from RSC-generated remosomes and slid nucleosomes. ^32^P 5’-labeled 255 bp 601 DNA fragment containing randomly incorporated uracil residues was used for reconstitution of centrally positioned nucleosomes. The nucleosomes were treated with RSC either in the absence of ATP (control particles) or in the presence of ATP to produce ∼ 50% mobilized particles. The remodeling reaction was arrested and the samples were separated on native PAGE. The end-positioned slid nucleosomes and the non-mobilized nucleosomes (containing the remosome fraction) as well as the control nucleosomes were eluted from the gel slice. The particles were then treated with the indicated increasing concentrations of UDG, the cleaved DNA was isolated and run on 8% PAGE under denaturing conditions; DNA, naked 255 bp 601 DNA fragment digested with UDG; first and last lane, •OH footprinting of native nucleosomes; on the right part of the figure is shown a schematics of the reconstituted nucleosome.

Since FACT significantly increases the capacity of RSC to generate remosomes and slid nucleosomes, its involvement in repair might be mainly associated with this property. If this was the case, one should expect damaged remosomal DNA to be easily repaired. We have addressed this question by studying the ability of UDG to remove uracil from remosomes (Fig 5). In agreement with the data in Fig 2, UDG was unable to excise histone octamer facing uracil from the control nucleosome (Fig 5, “nucleosomes”). In contrast, the histone octamer facing uracil was rather efficiently removed in remosomes, even at our lowest concentration of UDG (3.9x10^-3^ units), just like solution facing uracil (Fig 5, “remosomes”) and is almost saturated at concentrations above ∼2.3x10^-2^ units of UDG (Fig 5, “remosomes”). Excision of uracil from end positioned slid nucleosomes exhibits essentially the same behavior (Fig 5, “slid”). These results reveal that the alterations of either the histone-DNA interactions in the remosomes or mobilization of the nucleosomes allow efficient repair.

### The presence of FACT increases the efficiency of RSC to transform the energy freed by ATP hydrolysis into “mechanical” work

**Fig 6:**
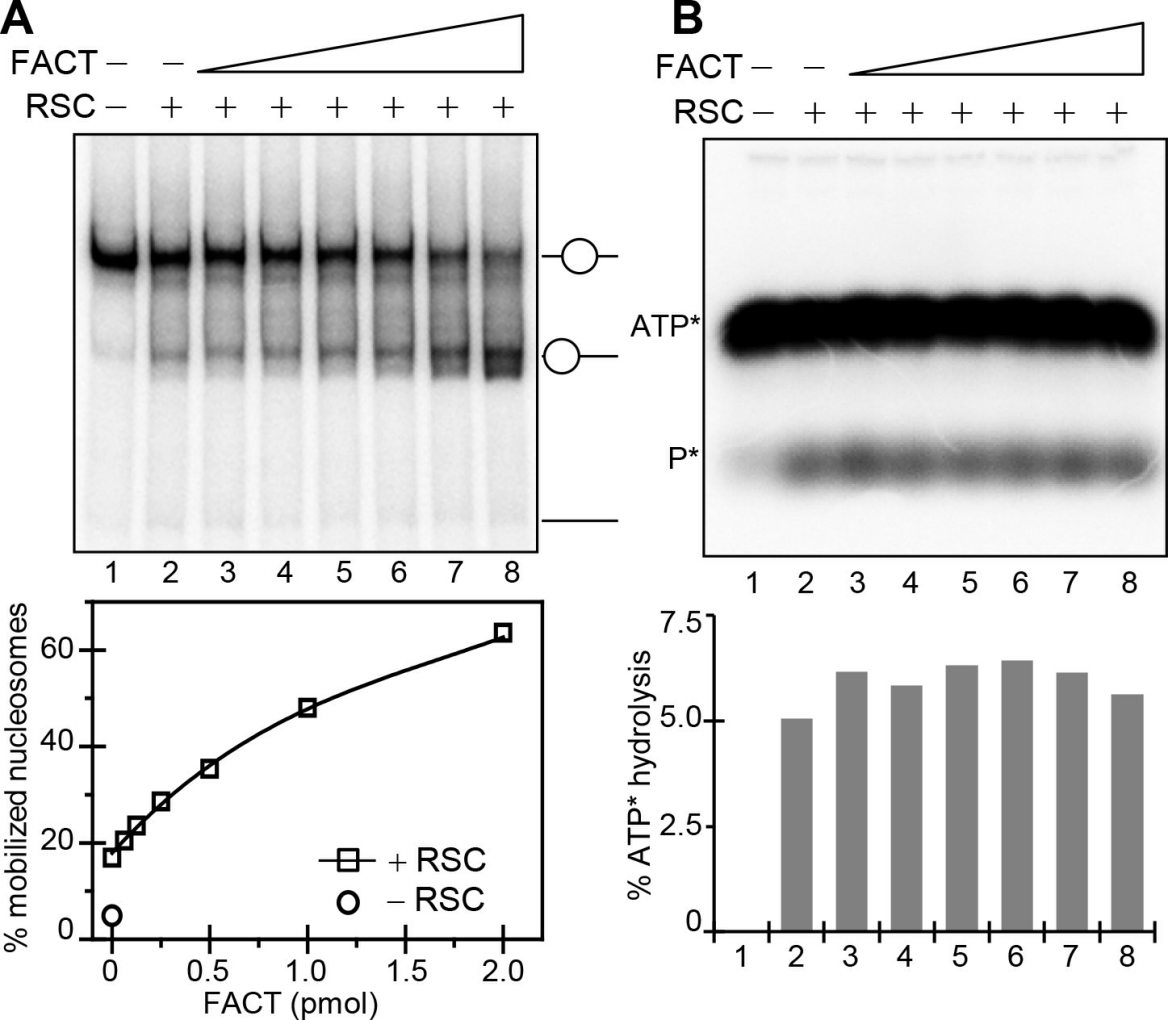
FACT increases the efficiency of nucleosome remodeling by RSC without affecting the ATP hydrolysis. (**A**) Nucleosome mobilization assay. Centrally positioned nucleosomes were incubated with 0.3 units of RSC at 120 μM of ATP in either the absence or presence of increasing amount of FACT for 50 minutes at 30°C. After arresting the reaction, the samples were run on native PAGE. The bands corresponding to the centrally and end-positioned nucleosomes are indicated. The lower panel represents the respective quantified data. (**B**) ATPase hydrolysis assay. Centrally positioned nucleosomes were incubated with RSC (0.3 units) and increasing amount of FACT in the presence of 120 μM of ATP and 3.3 μM of ^32^P-γATP. The products of the ATP hydrolysis were analyzed on 15% PAGE under denaturing conditions. Lower panel shows the respective quantified data. Fluctuations of the values are within the experimental error, typically ±10%.

The above presented data reveals that FACT assists very efficiently RSC to both alter the histone-DNA interaction and to mobilize the nucleosome in an ATP-dependent manner. To achieve this, FACT could either act on the nucleosomal substrate or on RSC or on both of them. To differentiate between these possibilities we have carried out the RSC nucleosome mobilization assay at increasing concentration of FACT, but at low ATP concentration (120 μM). At this low concentration of ATP it is possible to precisely measure the amount of ATP hydrolyzed by RSC and thus, to precisely determine the percentage of nucleosomes mobilized by the hydrolysis of a “unit” of ATP. Under these conditions of the experiment, the increase of the FACT concentration results in a ~ 4 fold increase (from 16% to 64%) of the slid by RSC nucleosomes (Fig 6A). Remarkably, under the same experimental conditions no change in the amount of hydrolyzed ATP was detected (Fig 6B). Kinetic experiments confirmed that FACT does not affect the ATPase activity of RSC (S3 Fig). This demonstrates that FACT boosts strongly the generation of slid nucleosomes (∼4-times more slid nucleosomes are generated per unit of hydrolyzed ATP in the presence of the highest amount of FACT (1.6 pmol) used in the experiments) without affecting the ATPase activity of RSC. In other words, the presence of FACT allows RSC to transform much more efficiently the energy freed by the ATP hydrolysis into mechanical work. Since FACT does not affect the ATPase activity of RSC, it should act on the nucleosomes making them more prone to remodeling.

## Discussion

Several reports clearly demonstrated the involvement of FACT in assisting the transcription of chromatin DNA [33,34,51,52]. The reported data on the potential implication of FACT in DNA repair are, however, scarce and no data on implication of FACT in oxidative damage repair are available [35–37]. Here we show that FACT is directly implicated in the repair of oxidatively generated DNA lesions and decipher how FACT may function in BER.

Our *in vivo* data reveals that upon oxidative stress FACT is released from transcribed chromatin and it is relocated to chromatin loci associated with both repair proteins and chromatin remodelers involved in DNA repair. This intriguing behavior of FACT points to a possible direct function in BER. To address this we have analyzed *in vitro* the effect of FACT on BER initiation by using chromatinized templates containing uracil. Our data shows that FACT alone has no effect on the removal of uracil by UDG from nucleosomal DNA. Although, FACT exhibits a strong “co-remodeling” activity and it is able to increase many-fold the efficiency of the involved in DNA repair chromatin remodeler RSC to both remodel and relocate the nucleosomes. FACT does not affect the ATPase activity of RSC, but instead makes the nucleosomes easier to be remodeled and mobilized, i.e. it increases the efficiency of transformation of the energy freed by the RSC-induced hydrolysis of ATP into mechanical work. This allows, in turn, very low amount of RSC to be sufficient to strongly alter the histone-DNA interactions as well as to slide the nucleosomes and thus, the lesions in chromatin DNA to be efficiently repaired. Our data suggest that *in vivo* FACT acts in BER, via RSC (or through other ATP-dependent remodeling factors), by increasing the efficiency of repair of DNA lesions.

FACT (at the concentrations used in the experiments) does not affect nucleosome structure as judged by both DNase I footprinting and the very sensitive restriction enzyme accessibility assay (one pot assay). In addition, no stable binding of FACT to the nucleosome was detected by EMSA. Then how does FACT act on the nucleosome substrate to make it more easily “remodelable”? It is difficult to answer to this question. Obviously, some transient FACT-nucleosome interactions during the RSC remodeling process should be generated allowing the remodeler to function with much higher efficiency. Noteworthy, genetic analysis in yeast has revealed evidences for functional relationship between the N-terminal domain of Spt16, one of the FACT subunits, and the docking domain of H2A [53]. The proper folding and integrity of the docking domain of H2A is, however, required for chromatin remodeling [54]. For example, nucleosome reconstituted with deleted docking domain H2A or with the histone variant H2A.Bbd (which possesses defective docking domain) cannot be both remodeled and mobilized by remodelers from both SWI/SNF and ISWI family [54,55]. Of note, Hondele et al., have provided structural insights into Spt16 binding to H2A-H2B dimers [56] and suggested a possible mechanism of unraveling of the outer 30 base pairs of the nucleosome by invasion of FACT. Although, it has been reported that FACT alone is able to alter the overall nucleosome structure and destabilize both histone-DNA and histone-histone interactions without any histone eviction [53]. In these last experiments the molar ratio FACT/nucleosome exceeded, however, at least 50 fold the ratio of FACT/nucleosome used in our experiments (see Materials and Methods). Thus, the reported FACT-induced destabilization of the nucleosome would reflect the very high concentrations of FACT in the earlier reported experiments [53]. Displacement or transfer of histone dimers as a result of nucleosome remodeling has been reported [57] indicative of destabilization of nucleosomes in the process. As mentioned above, H2A variants or experimental mutations which weaken the H3-H4 tetramer and H2A-H2B dimer interface result in unwrapping of DNA from the nucleosomal ends and associated loss of remodeling. An attractive possibility is that depending on the local concentration of FACT, the equilibrium can be shifted into a ‘dynamically stable’ conformation of nucleosomes, which facilitates efficient nucleosome remodeling.

It has been recently shown that at the sites of UV induced damage, the histone pair H2A-H2B is exchanged and this exchange is assisted by FACT. This FACT-dependent chromatin dynamics is implicated in the promotion of transcriptional restart after DNA repair of the lesions blocking transcription [38]. Thus, in Transcription-Coupled–Nucleotide Excision Repair (TC-NER), the histone chaperone activity of FACT appeared to be used. This might reflect the nature of lesions to be repaired requiring the removal of histones to freeing-up a relatively long stretch of free DNA for TC-NER to proceed efficiently.

Of note, the observed co-remodeling activity of nucleolin, a protein displaying histone chaperone activity [44], suggests that RSC boosting could be a common feature for histone chaperones. However, under our experimental conditions we did not observe any SWI/SNF and RSC remodeler activity boosting mediated by some “classical” histone chaperones we tested, namely NPM1 (B23), mNAP1, and nucleoplasmin (S4 Fig). Since both FACT [33] and nucleolin [58] contains an HMG box domain and HMGB1&2 proteins have also co-remodeling activity [59], this suggests that the presence of HMG-box would be important for co-remodeling.

Interestingly, FACT facilitates also very efficiently the nucleosome mobilization by ACF (S5 Fig), a remodeler belonging to the ISWI family. FACT was also found stably recruited to the CENP-A nucleosomal complexes [60] and RSF, a chromatin remodeler, is also a member of this complex [61,62]. In addition, FACT was shown to physically interact with CHD1, another chromatin remodeler [63]. Thus, the cell appears to use the FACT nucleosome reorganization ability to control the activity of distinct remodelers belonging to different families for different purposes in different processes. The transient interaction of FACT with H2A docking domain and with nucleosomal DNA would be determinant for this peculiar property of FACT and the cell may use it not only to modulate the remodeling capacity of chromatin remodeling machines but also to control other “machines” working on chromatin. A typical example is the activity of FACT during transcription, which requires a transient interaction with the H2A/H2B dimer [52].

## Materials and Methods

### Preparation of DNA fragments

The 255 bp 601 DNA probe used for reconstitution of centrally positioned nucleosomes was PCR amplified from pGEM-3Z-601.1 plasmid (kindly provided by J. Widom). 5’ end labeling was performed by using γ-^32^P-labeled primer in PCR. For ‘One Pot Restriction enzyme Assay’ a set of eight pGEM-3Z-601.2 mutants were utilized, each containing *Hae*III site at a different superhelical location, as described before [50]; note that the “dyad 7” fragment contains an additional *Hae*III site located at 4 bp away from the d7 site). Briefly, a 281 bp fragment was amplified using primers targeting the vector specific sequence flanking the 601.2 sequence. Labeling of the fragment was done as described above. The fragments were subsequently digested with *Sph*I to get a fragment of 255 bp with 57 and 51 bp linker DNA on left and right side respectively. For repair assays uracil was randomly incorporated in the 601 sequence by PCR using a dNTP mix containing dUTP/dTTP in a 5/95% ratio [30]. Top strand was 5’ end labeled using γ-^32^P-labeled primer in PCR as described. For DNase I footprinting, a 200 bp fragment with 601 positioning sequence at one end was obtained by digestion of 255 bp 601 with NotI. Fragments were labeled by gap filling using Klenow enzyme with [α-^32^P]CTP in the presence of 50 μM dGTP. For end positioned nucleosomes used for ACF induced nucleosome sliding assays, a 241 bp fragment was PCR amplified from p199.1 plasmid to generate 601 DNA with the 601 sequence at the end and labeled using γ-^32^P-labeled primer in PCR. All the DNA fragments were purified on 6% native acrylamide gel prior to use for nucleosome reconstitutions.

### Proteins

The chromatin associated FACT complex was affinity purified from HeLa cell extracts as described previously [64]. Recombinant histones H2A, H2B, H3 and H4 from *Xenopus laevis* were expressed in *Echerichia coli* strain BL21 (DE3) and purified to homogeneity under denaturing conditions as described [65]. *Saccharomyces cerevisiae* RSC complex was purified using standard tandem affinity purification as described [24]. ACF remodelling complex was purified according the protocol described in [55]. APE1 is from NEB and UDG from Invitrogen.

### Purification of e-FACT complex

Oxidative DNA lesions were generated by HeLa cells incubation on ice for 5 min with 10 mM H_2_O_2_. HeLa extracts were prepared using a modification of the Dignam protocol [66]. Briefly, cells were lysed in hypotonic buffer (10 mm Tris-HCl at pH 7.65, 1.5 mm MgCl_2_, 10 mm KCl) and disrupted by Dounce homogenizer. The cytosolic fraction was separated from the pellet by centrifugation at 4°C. The nuclear-soluble fraction was removed by incubation of the pellet in high-salt buffer (to get a final NaCl concentration of 300 mM). Nuclear pellets were recovered by centrifugation and resuspended in TGEN 50 buffer (20 mM Tris at pH 7.65, 50 mM NaCl, 3 mM MgCl_2_, 0.1 mM EDTA, 10% glycerol, 0 0.01% NP40). Nuclei were digested by micrococcal nuclease to generate mainly mononucleosomes. Mononucleosomes containing e-SSRP-1 were purified from the resulting material by immunoprecipitation on anti-Flag antibody-conjugated agarose. After elution with the Flag peptide, the e-SSRP-1 bound nucleosomes were incubated with 1M NaCl and further fractionated on a 15–45% glycerol gradient to dissociate FACT from the nucleosome. The e-SSRP-1 complex was further affinity-purified by anti-HA antibody-conjugated agarose and eluted with the HA peptide. The HA and Flag peptides were first buffered with 50 mM Tris-Cl (pH 8.5), then diluted to 4 mg/mL in TGEN 150 buffer (20 mM Tris at pH 7.65, 150 mM NaCl, 3 mM MgCl_2_, 0.1 mM EDTA, 10% glycerol, 0 0.01% NP40), and stored at −20°C until use. Anti-FLAG beads were washed in TGEN 150 buffer. Complexes were resolved by SDS-PAGE and stained using the Silver Quest kit (Invitrogen). Identification of proteins was performed by the Taplin Biological Mass Spectrometry Facility (Harvard Medical School, Boston, Massachusetts, USA).

### Photoinduction of DNA lesions

Damage to DNA was induced mainly as described in [67]. HeLa cells transfected with the indicated Fusions with either DsRed or EGFP were grown on coverslips in DMEM medium and were treated with Ro-198022 for 5 min before 405 nm laser irradiation. The irradiation was performed at low intensity generating no DSB and UV-lesions as described in [39].

### Reconstitution of nucleosomes

Nucleosomes were reconstituted using the salt dialysis procedure (Mutskov et al., 1998, MCB). Briefly, 5 μg of chicken erythrocyte carrier DNA and 250 ng of abovementioned labeled DNA probes were mixed with pre-reconstituted core histone octamers in an equimolar ratio. The nucleosome reconstitution buffer was composed of 10 mM Tris–HCl (pH 7.4), 1 mM EDTA, 5mM β- mercaptoethanol and 2 M NaCl. Reconstitution mixtures were dialyzed at 4°C against a smoothly decreasing NaCl concentration up to 10 mM final. Efficiency and quality of nucleosome reconstitutions were checked on a 5% native PAGE run with 0.25X TBE.

### Nucleosome remodeling assays

Nucleosome sliding reactions were performed typically with ~75 ng of nucleosomes (~0.5 pmol) and the indicated amount of RSC or ACF in remodeling buffer (RB) consisting of 10 mM Tris pH 7.4, 5% glycerol, 1 mM ATP, 2.5 mM MgCl_2_, 1 mM DTT, 100 μg/ml BSA, 50 mM NaCl, 0.01% NP40 in a volume of 7.5 μl at 29°C for 50 min. Reactions were stopped by addition of 0.1 units of apyrase. Sliding products were analysed, when indicated, on a 5% native PAGE by quantifying the exposed autoradiographs. Similar conditions were used for remodeling assays to be probed by DNase I or followed by repair assays if not mentioned otherwise. One unit of the remodeling complexes is defined as the amount (typically ~10 fmol) necessary to induce 50% relocation to the end position of ~0.5 pmol middle positioned 601 ~250 bp nucleosomes in 50 min under our standard reaction conditions [14,31,44].

### DNase I and hydroxyl radical footprinting

One μg of plasmid DNA was added to the end positioned control or remodelled nucleosomes after stopping the reaction by addition of apyrase. Samples were digested for 2 min with 0.5 units of DNase I at room temperature. After stopping the reaction by addition of SDS and EDTA to 0.1% and 20 mM respectively, DNA was extracted with phenol-chloroform, ethanol precipitated and run on 8% denaturing PAGE. Dried gels were exposed and imaged on Fuji-FLA5100 phosphorimager.

Nucleosomes (0.5 pmol) assembled on the end labelled 255 bp 601 DNA were adjusted to 5 mM Tris (pH 7.4) and 0.25 mM EDTA in a 7.5 μl volume. The hydroxyl radical •OH reaction was carried out by mixing 2.5 μl each of 2 mM FeAMSO₄ and 4 mM EDTA, 1M ascorbate and 0.12% H_2_O_2_ in a drop on the side of the tube cap before mixing it with the nucleosome solution. The reaction was stopped by adding 0.1% SDS, 20 mM EDTA, 1% glycerol and 100 mM Tris (pH 7.4). DNA was purified and analyzed as described above.

### BER initiation assays

For carrying out the repair assay the DNA or nucleosomes incorporated Uracil were incubated with indicated amount of Uracil DNA glycosylase (UDG) in the remodeling buffer at 30°C for one hour. DNA was phenol chloroform extracted and precipitated with ethanol. Abasic sites generated by UDG were cleaved by incubation with 1 unit of APE1. DNA was extracted, processed and imaged as described above.

### In gel one pot assay

A sliding reaction was carried out in the presence of increasing concentrations of FACT and the amounts of RSC as indicated. Prior to loading on 5% native polyacrylamide gel, 6.25 pmol of cold 255 bp 601 middle positioned nucleosomes were added to each reaction as a carrier in order to maintain stability during subsequent procedures. Remodeling products were resolved on 5% native polyacrylamide gel. Bands, corresponding to control unremodeled and unmobilized remodeled nucleosomes, were excised, collected in siliconized eppendorf tubes, crushed very gently and immersed with 50 μl restriction buffer (10 mM Tris pH 7.6, 10 mM MgCl_2_, 50 mM NaCl, 1 mM DTT and 100 μg/ml BSA) containing 8 units/μl of *Hae*III for 10 minutes at 30°C. The reaction was stopped by adding an equal volume (50 μl) of 2x stop buffer containing 0.2% SDS and 40 mM EDTA. DNA was eluted from the gel slices, purified as described above, and run on 8% denaturing gel. The quantification of extent of accessibility at different superhelical locations in the nucleosome was performed using Multi-Gauge Software (Fuji) as described [24].

### Gel purification of nucleosomes, remosomes and slid fractions

Centrally positioned 601 nucleosomes were incubated for 50 min with RSC in the remodeling reaction as described above to achieve ~50–60% siding [24]. Reaction products were resolved on 5% native polyacrylamide gel. Bands, corresponding, the un-mobilized RSC remodeled “remosomes”, and the mobilized end-positioned “slid” fractions, as well as control naked DNA and untreated nucleosomes were excised. Excised bands were eluted in 80 μl Elution Buffer (EB) containing Tris 10 mM pH 7.4, 0.25 mM EDTA, 0.01% NP40, 100 μg/ml BSA and 30 mM NaCl, at 4°C for 1 h with gentle shaking. 7.5 pmol of cold 255 bp 601 nucleosomes were added in the elution buffer to maintain the stability of eluted nucleosomes. Eluted nucleosomes were filtered through glass fibre filter under low speed centrifugation (200g) to remove acrylamide particles.

Eluted naked DNA, nucleosomes, remosomes and slid particles were divided into equal aliquots, and uracil cleavage efficiency was assessed by incubating the fractions with UDG in increasing concentrations. Samples were processed for further analysis as described above.

### ATPase assay

The ATPase assays were carried out in the remodeling reaction buffer with ATP concentration at 120 μM. To the reaction mixture 0.1 μl (3.3 μM) of the source ^32^P ATP was added. The remodelling reaction was carried out at 29°C for 50 minutes. Reaction aliquots were analyzed by both, native 5% PAGE (sliding assay) and 15% acrylamide-50% urea denaturing gel (ATPase assay). The native PAGE was dried, exposed overnight on a phosphor imager screen and quantified by using the Multi Gauge V3.0 software. The wet denaturing gels (ATPase assay) were covered by saran, exposed for 30’ and the digital images quantified. Briefly, %ATP_hyd_ = 100[P*]/([P*]+[ATP*]) (where the values in brackets represents the background corrected integrated volumes of the corresponding radioactive bands on the digital gel image. The experimental error was typically ±10% from two independent replicas.

## Supporting Information

S1 Fig Characterization of the reconstituted nucleosomal particles. (**A)** SDS electrophoresis of purified recombinant histones and the histone octamer. (**B**) EMSA of the 255 bp 601 DNA (left) and reconstituted centrally positioned nucleosomes (right). (**C**) SDS electrophoresis of hFACT. Positions of the two subunits of FACT (Spt16 and SSRP1) are indicated.
(TIF)

S2 Fig Effect of FACT on the time course of RSC-induced nucleosome mobilization. **(A)** Time course of nucleosome mobilization in the absence (left panel) or in the presence (right panel) of FACT. Centrally positioned 601 nucleosomes were incubated with 1 unit of RSC and 1.6 pmol of FACT for the times indicated at 30°C in the presence of 1 mM ATP. After arresting the reaction, repositioning of the nucleosomes was analyzed by native EMSA.
(TIF)

S3 Fig FACT facilitates ACF nucleosome mobilization. (A) EMSA of ACF induced nucleosome mobilization in the presence of increasing amount of FACT. End-positioned 601 nucleosomes were incubated with 0.2 units of ACF either in the absence (lane 3) or in the presence of increasing concentration of FACT (lanes, 4–9). After arresting the reaction, the reaction products were run on a native PAGE; lane 10, EMSA of the nucleosomes incubated with 2 units of ACF in the absence of FACT; lanes 1 and 2, controls showing the input nucleosomes and incubated with FACT nucleosomes in the absence of ACF, respectively. All reaction solutions contained 1 mM ATP. (B) Quantification of the data presented in (A).
(TIF)

S4 Fig Nucleolin and FACT, but not mNAP1, NPM1 and nucleoplasmin, enhance SWI/SNF-induced remodeling of nucleosomes. End-positioned 241 bp 601 nucleosomes were incubated for 50 min at 30°C with 0.2 (or 1.0) units of SWI/SNF in the absence or in the presence of 2.0 pmol of histone chaperone as indicated. The remodeling reactions were arrested, and the DNA digested with 0.1 units of DNase I for 2 min. The cleaved DNA was purified and analyzed by 8% sequencing PAGE under denaturing conditions.
(TIF)

S5 Fig The ATPase activity of RSC is independent on FACT: time-course experiment. Centrally positioned 601 nucleosomes were incubated with 1.5 units of RSC for the times indicated at 30°C in the absence (left panel) or the presence (right panel) of 1.6 pmol of FACT in standard buffer containing 80 μM of ATP and 2.2 μM of ^32^P-γATP. The products of the ATP* hydrolysis were analyzed on 15% denaturing PAGE. Control data are also shown. Lower panel shows the respective quantified data. The experimental error is typically ±10%.
(TIF)

S1 Table Mass spectrometry identification of the polypeptides associated with control FACT chromatin bound complex (-) or with the FACT chromatin bound complex, isolated from H_2_O_2_ treated cells (+). Proteins present in the e-SSRP1.com together with the number of identified peptides are indicated. Proteins involved in transcription are shown in red. DNA repair proteins and chromatin remodelers are shown in blue. The different proteins which exhibited similar number of identified peptides in the e-SSRP1.com from control and H_2_O_2_ treated cells are shown in black.
(DOCX)
