# Supplementary figures and images for: FACT Assists Base Excision Repair by Boosting the Remodeling Activity of RSC
Source: PLoS Genet. 2016 Jul 28;12(7):e1006221. doi: 10.1371/journal.pgen.1006221 (PMC4965029; doi:10.1371/journal.pgen.1006221)

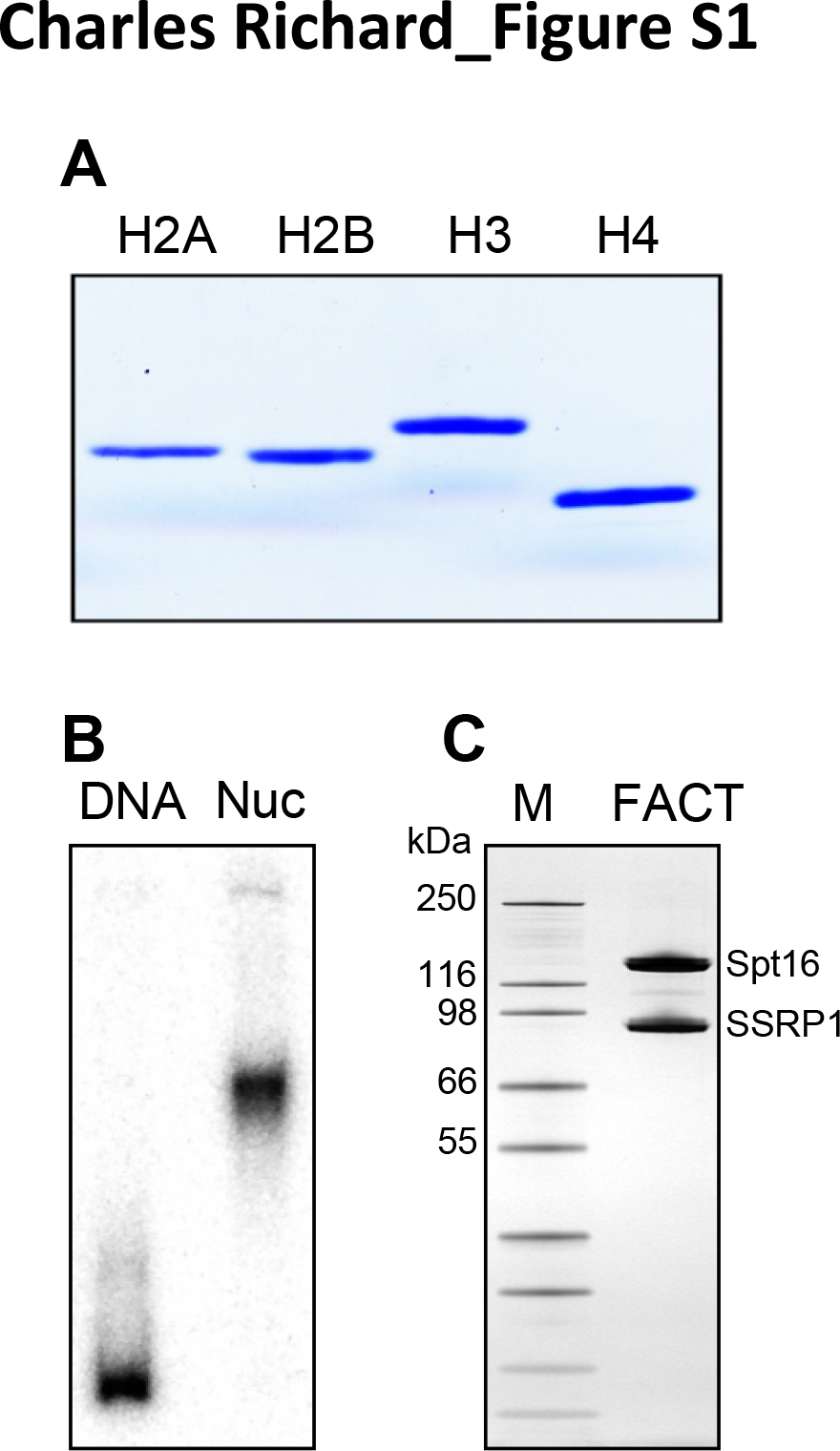

Supplement: S1 Fig — (A) SDS electrophoresis of purified recombinant histones and the histone octamer. (B) EMSA of the 255 bp 601 DNA (left) and reconstituted centrally positioned nucleosomes (right). (C) SDS electrophoresis of hFACT. Positions of the two subunits of FACT (Spt16 and SSRP1) are indicated. (TIF) [file pgen.1006221.s001.tif]

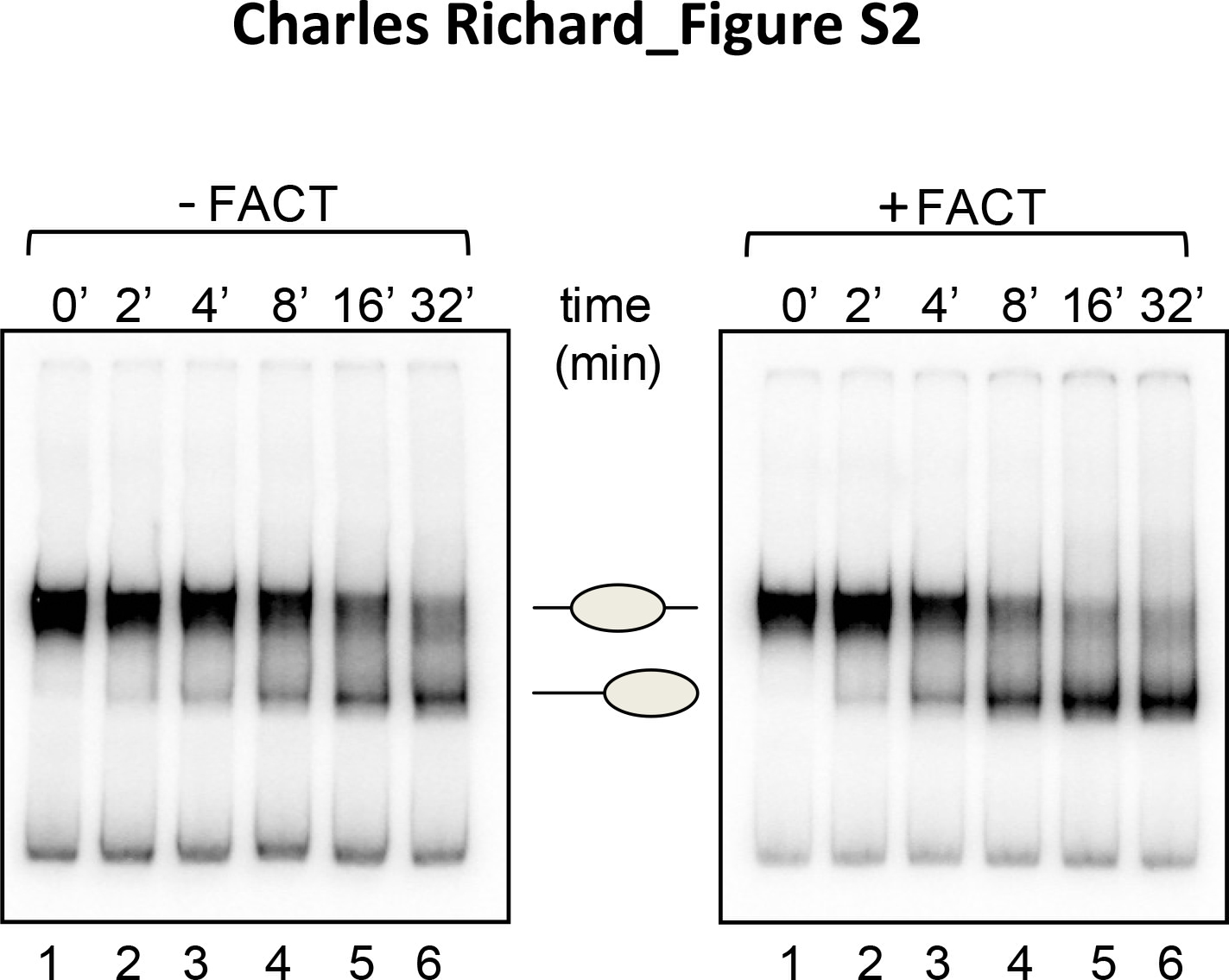

Supplement: S2 Fig — (A) Time course of nucleosome mobilization in the absence (left panel) or in the presence (right panel) of FACT. Centrally positioned 601 nucleosomes were incubated with 1 unit of RSC and 1.6 pmol of FACT for the times indicated at 30°C in the presence of 1 mM ATP. After arresting the reaction, repositioning of the nucleosomes was analyzed by native EMSA. (TIF) [file pgen.1006221.s002.tif]

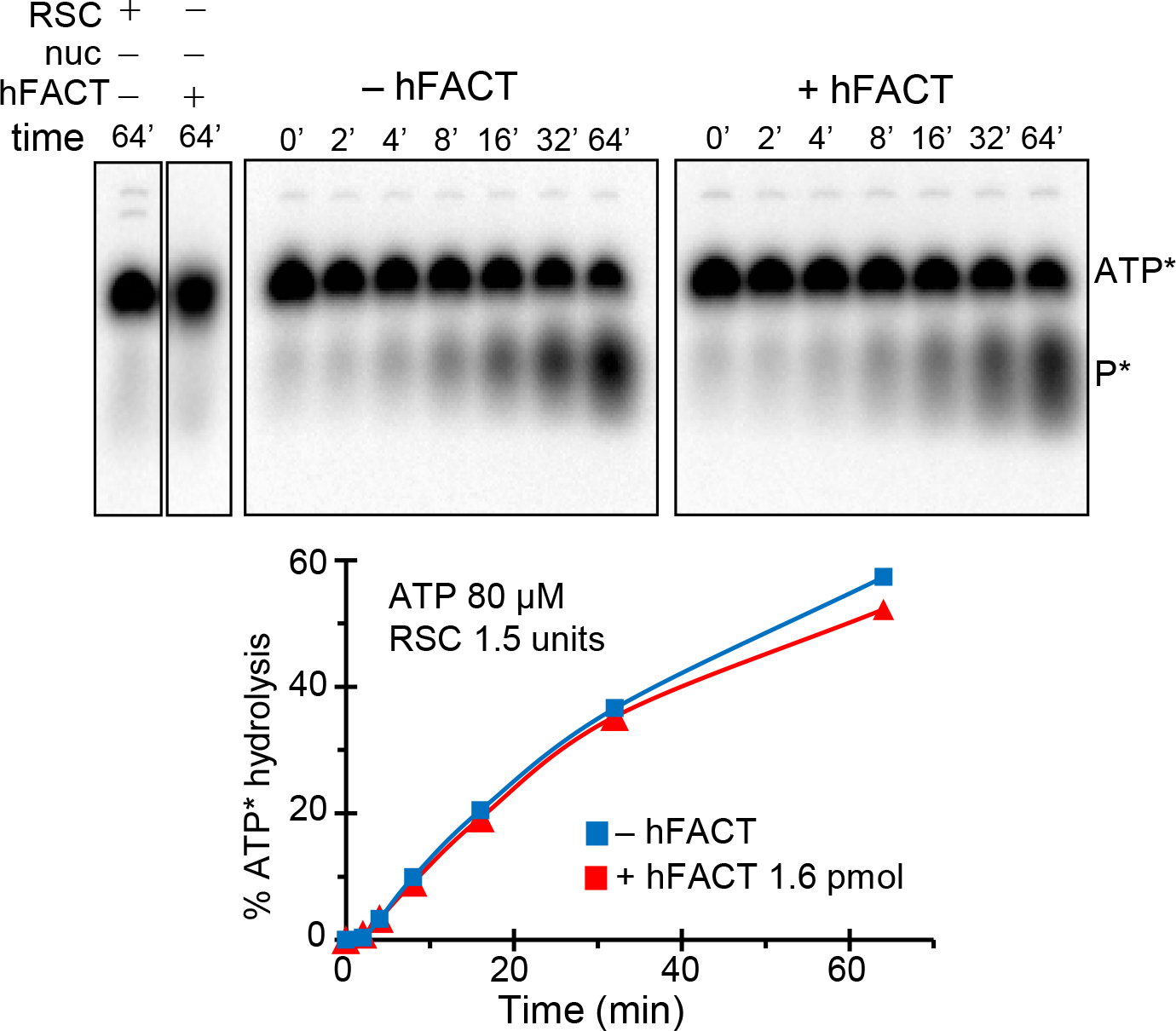

Supplement: S3 Fig — (A) EMSA of ACF induced nucleosome mobilization in the presence of increasing amount of FACT. End-positioned 601 nucleosomes were incubated with 0.2 units of ACF either in the absence (lane 3) or in the presence of increasing concentration of FACT (lanes, 4–9). After arresting the reaction, the reaction products were run on a native PAGE; lane 10, EMSA of the nucleosomes incubated with 2 units of ACF in the absence of FACT; lanes 1 and 2, controls showing the input nucleosomes and incubated with FACT nucleosomes in the absence of ACF, respectively. All reaction solutions contained 1 mM ATP. (B) Quantification of the data presented in (A). (TIF) [file pgen.1006221.s003.tif]

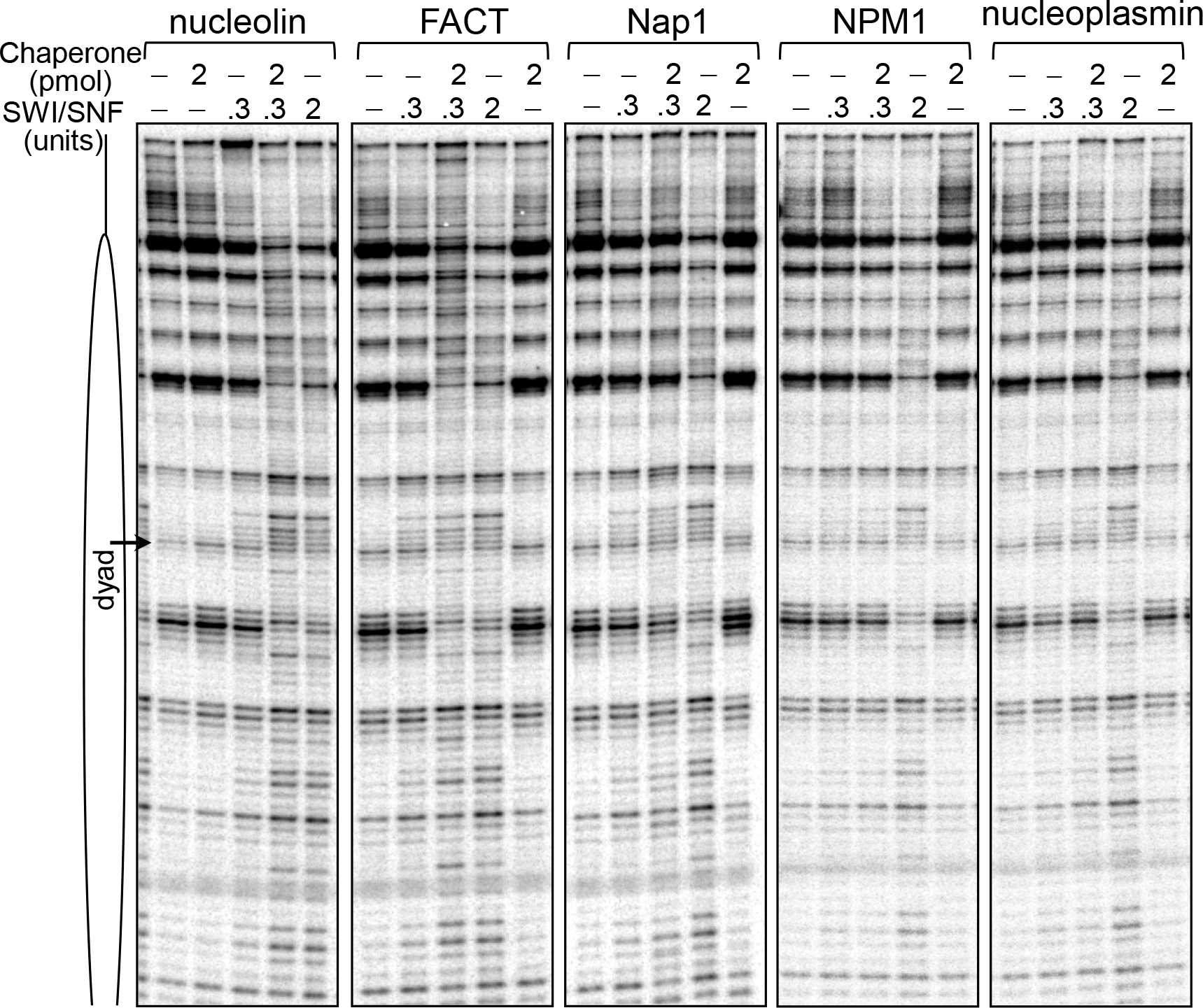

Supplement: S4 Fig — End-positioned 241 bp 601 nucleosomes were incubated for 50 min at 30°C with 0.2 (or 1.0) units of SWI/SNF in the absence or in the presence of 2.0 pmol of histone chaperone as indicated. The remodeling reactions were arrested, and the DNA digested with 0.1 units of DNase I for 2 min. The cleaved DNA was purified and analyzed by 8% sequencing PAGE under denaturing conditions. (TIF) [file pgen.1006221.s004.tif]

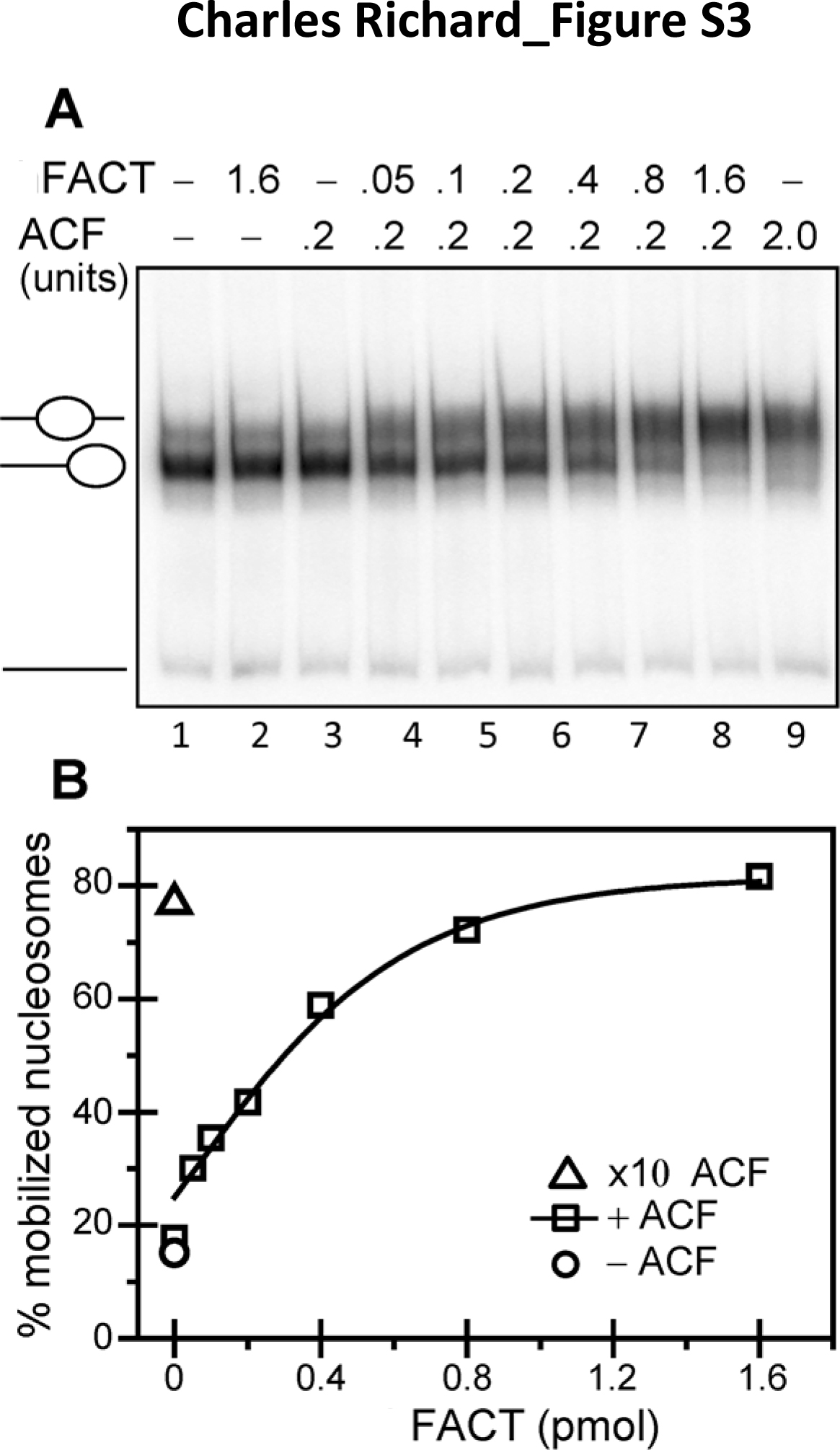

Supplement: S5 Fig — Centrally positioned 601 nucleosomes were incubated with 1.5 units of RSC for the times indicated at 30°C in the absence (left panel) or the presence (right panel) of 1.6 pmol of FACT in standard buffer containing 80 μM of ATP and 2.2 μM of 32P-γATP. The products of the ATP* hydrolysis were analyzed on 15% denaturing PAGE. Control data are also shown. Lower panel shows the respective quantified data. The experimental error is typically ±10%. (TIF) [file pgen.1006221.s005.tif]
