# Supplementary material for: FACT Assists Base Excision Repair by Boosting the Remodeling Activity of RSC
Source: PLoS Genet. 2016 Jul 28;12(7):e1006221. doi: 10.1371/journal.pgen.1006221 (PMC4965029; doi:10.1371/journal.pgen.1006221)
Supplement: S1 Table — Proteins present in the e-SSRP1.com together with the number of identified peptides are indicated. Proteins involved in transcription are shown in red. DNA repair proteins and chromatin remodelers are shown in blue. The different proteins which exhibited similar number of identified peptides in the e-SSRP1.com from control and H2O2 treated cells are shown in black. (DOCX) [file pgen.1006221.s006.docx]

**Charles Richard_Table S1**

| Proteins | Access. N° | -H_2_O_2_ | +H_2_O_2_ |  | Proteins | Access. N° | -H_2_O_2_ | +H_2_O_2_ |
| --- | --- | --- | --- | --- | --- | --- | --- | --- |
| POLR2A | P24928 | 15 | 0 |  | SNRNP200 | O75643 | 26 | 32 |
| POLR2B | P30876 | 14 | 0 |  | PRPF8 | Q6P2Q9 | 21 | 35 |
| PAF1 | B4DGJ5 | 4 | 0 |  | PLEC1 | Q15149 | 21 | 18 |
| HIRIP3 | Q9BW71 | 11 | 0 |  | HNRNPU | Q00839 | 25 | 30 |
| TRIM24 | O15164 | 13 | 0 |  | SF3B1 | O75533 | 14 | 14 |
| TRIM33 | Q9UPN9 | 8 | 0 |  | SF3B2 | Q13435 | 18 | 16 |
| DDB1 | Q16531 | 0 | 20 |  | SF3B3 | Q15393 | 16 | 10 |
| DDB2 | Q92466 | 0 | 4 |  | TUBB2C | P68371 | 22 | 25 |
| PolD1 | P28340 | 0 | 18 |  | SMC1A | Q14683 | 16 | 13 |
| SMARCA4 | P51532 | 0 | 18 |  | SMC3 | Q9UQE7 | 17 | 13 |
| SMARCA1 | P28370 | 0 | 8 |  | HSPA8 | P11142 | 18 | 13 |
| SMARCC2 | Q8TAQ2 | 0 | 11 |  | HNRNPM | P52272 | 15 | 13 |
| MRE11A | P49959 | 0 | 5 |  | EFTUD2 | Q15029 | 14 | 18 |
| Rad50 | Q92878 | 0 | 8 |  | MATR3 | P43243 | 15 | 7 |
| LIG1 | P18858 | 0 | 3 |  | MSH6 | P52701 | 10 | 28 |
| LIG3 | P49916 | 0 | 8 |  | MSH2 | P43246 | 6 | 22 |
| UBA2 | Q9UBT2 | 0 | 6 |  | DHX9 | Q08211 | 10 | 26 |
| CUL4A | Q13619 | 0 | 11 |  | USP7 | Q93009 | 9 | 22 |
| CUL4B | Q13620 | 0 | 7 |  | TUBA4A | P68366 | 8 | 15 |
| XRCC4 | Q13426 | 0 | 8 |  | PRPF6 | O94906 | 8 | 5 |
| XRCC1 | P18887 | 0 | 7 |  | HNRNPR | O43390 | 8 | 12 |
| PARP1 | P09874 | 18 | 37 |  | DHX15 | O43143 | 8 | 19 |
| SUPT16H | Q9Y5B9 | 633 | 265 |  | HNRNPUL2 | Q1KMD3 | 8 | 13 |
| SSRP1 | Q08945 | 253 | 180 |  | H4 | P62805 | 108 | 99 |
| PRKDC | P78527 | 66 | 137 |  | H2B | Q16778 | 87 | 83 |
| TOP1 | P11387 | 33 | 16 |  | H2A | P04908 | 33 | 37 |
| XRCC5 | P13010 | 30 | 24 |  | H3 | Q71DI3 | 27 | 26 |
| XRCC6 | P12956 | 24 | 29 |  |  |  |  |  |
